# Electrocardiographic Abnormalities in Hospitalized Patients with COVID-19 and the Associations with Clinical Outcome

**DOI:** 10.3390/jcm11175248

**Published:** 2022-09-05

**Authors:** Francesco Carubbi, Alessia Alunno, Augusto Carducci, Davide Grassi, Claudio Ferri

**Affiliations:** 1 Department of Life, Health & Environmental Sciences, University of L’Aquila, 67100 L’Aquila, Italy; 2 Internal Medicine and Nephrology Unit, Department of Medicine, ASL Avezzano-Sulmona-L’Aquila, San Salvatore Hospital, 67100 L’Aquila, Italy

**Keywords:** COVID-19, ECG, cardiovascular disease

## Abstract

The cardiovascular (CV) system can often be affected during SARS-CoV-2 infection and several acute manifestations, such as myocardial infarction, pericarditis, myocarditis and arrhythmias have been described. We provide a retrospective overview of electrocardiographic (ECG) features and their relationship with clinical outcomes in a cohort of patients admitted to our COVID-19 Unit between November 2020 and May 2021. Resting standard 12-lead ECGs were performed in all patients at admission and in those recovering from SARS-CoV-2 infection also at discharge. Clinical and serological records alongside ECG measurements were retrospectively evaluated and statistical analysis was performed to identify relationships between variables. A total of 123 patients (44% females) with a mean age of 73.9 years were enrolled. Ninety-five (77%) patients recovered from SARS-CoV-2 infection and were discharged while 28 (23%) died in hospital. Almost 90% of patients displayed at least one CV risk factor and 41 (33%) patients had at least one previous CV event. We observed that heart rate, corrected QT interval dispersion (QTcd) and inverted T waves are independently associated with in-hospital death and inverted T waves show the strongest association. This association remained significant even after correcting for the number of CV risk factors at baseline and for the type of CV risk factor at baseline. Our study demonstrated that some ECG abnormalities at admission are independently associated with in-hospital death regardless of pre-existing CV risk factors. These findings may be of particular relevance in clinical settings with limited access to advanced techniques, such as cardiac magnetic resonance and could help improve the outcomes of patients with cardiac involvement related to SARS-CoV-2 infection.

## 1. Introduction

Severe acute respiratory syndrome coronavirus 2 (SARS-CoV-2) infection is characterized by a heterogeneous clinical picture ranging from no symptoms to respiratory failure, multi-organ damage and death. The cardiovascular (CV) system can often be affected during SARS-CoV-2 infection and several acute manifestations, such as myocardial infarction, pericarditis, myocarditis and arrhythmias have been described [1]. However, also long-term post-COVID-19 CV manifestations have been described [2,3] and an initial myocardial injury has been associated with increased mortality [4].

Several studies investigated the mechanisms underlying CV involvement during and after SARS-CoV-2 infection but they are not fully elucidated yet. A multifaceted pathogenic process encompassing direct viral cytopathic effects alongside local and systemic inflammatory damage and autoantibodies is the most likely scenario [5,6]. In particular, the abnormal inflammatory response and the excessive release of proinflammatory mediators leading to aberrant immune cell over-activation sets the stage for cell-mediated cytotoxicity by CD8+ T lymphocytes that migrate into the heart and cause myocardial inflammation [7].

This may explain not only the clinical manifestations observed during acute SARS-CoV-2 infection but also those observed after recovery from SARS-CoV-2 infection. In fact, long-term manifestations of the so-called “Long COVID” condition also include CV sequelae or new-onset CV manifestations that may depend on mechanisms other than inflammation, such as persistence of viral genome in the myocytes, the development of long-lasting autoantibodies [2] or structural acute and post-acute persistent changes in the myocardium after myocardial injury [8].

Other potential mechanisms may include electrolyte abnormalities during infection, and cardiac toxicity due to drugs. On top of infection-related events, drugs extensively used for the management of COVID-19 during the first and second waves, in particular, hydroxychloroquine with or without concomitant azithromycin and lopinavir-ritonavir, are able to elongate the QT interval, and therefore, facilitate ventricular arrhythmias [9].

On this basis, the aim of our study was to investigate electrocardiographic (ECG) features and their relationship with clinical outcomes in a cohort of patients admitted to our COVID-19 Unit.

## 2. Materials and Methods

### 2.1. Patient Cohort and Data Collection

Clinical and serological records of patients admitted to our COVID-19 Medicine Unit of the Abruzzo region in Italy (San Salvatore Hospital, L’Aquila, Italy) between November 2020 and May 2021, with clinical and laboratory signs of SARS-CoV-2 infection, as assessed by positive SARS-CoV-2 polymerase chain reaction method to detect the virus using a nasopharyngeal swab, were retrospectively evaluated. The patients did not receive any treatment for suspected SARS-CoV-2 infection prior to hospital admission. Collected data included: age, gender, days of symptoms before hospital admission, red blood cell count, hemoglobin (Hb), platelet (PLT) count, white blood cell (WBC) count, absolute neutrophil count (ANC), absolute lymphocyte count (ALC), absolute monocyte count (AMC), neutrophils:lymphocytes (N:L) ratio, erythrocyte sedimentation rate (ESR), C-reactive protein (CRP), ferritin, D-dimer, fibrinogen, lactate dehydrogenase (LDH), procalcitonin, arterial oxygen partial pressure:fractional inspired oxygen ratio (PaO2/FiO2). The following CV disease risk factors were considered: smoking (defined as previous/current/no use of at least one cigarette/day), arterial hypertension (physician diagnosis and/or prior/ongoing antihypertensive therapy), hypercholesterolemia (total serum cholesterol level > 200 mg dL−1 in at least three assays), hypertriglyceridemia (serum triglyceride level > 150 mg dL−1 in at least three assays), high-density lipoprotein cholesterol (HDL-c) level (reduced <40 mg dL−1, normal 40–60 mg dL−1, increased >60 mg dL−1 in at least three assays), low-density lipoprotein cholesterol level (increased >115 mg dL−1 in at least three assays), type 2 diabetes mellitus (DM) (ongoing treatment with insulin or oral hypoglycemic agents and/or glucose level > 126 mg dL−1 in at least two fasting glycemia tests) and obesity (according to body mass index). The degree of obesity was established as follows: 25 ≤ 30 kg/m^2^ (overweight), 30–34.9 kg/m^2^ (grade I obesity), 35–39.9 kg/m^2^ (grade II obesity), and ≥40 kg/m^2^ (grade III obesity or severe obesity), respectively. Metabolic syndrome was defined according to the National Cholesterol Education Program (NCEP) Adult Treatment Panel III (ATP III) criteria [10]. CV events, namely myocardial infarction, angina, heart failure, and cerebrovascular events, were recorded. We also considered arteriosclerotic vascular disease. CV events were recorded only if the diagnosis was confirmed by hospital discharge records and/or available specific laboratory and diagnostic examinations.

### 2.2. Electrocardiography

Resting standard 12-lead ECGs were performed using Mortara ELI 250c electrocardiograph machine in all patients at admission and in those recovering from SARS-CoV-2 infection also at discharge using a paper speed of 25 mm/s and a sensitivity of 1 mV = 10 mm. The following measures were calculated manually by two expert physicians (FC and DG) unless automatically calculated by the machine (disagreement was resolved by consulting a third expert (CF): heart rate (HR), PR interval, QT interval (along with minimum and maximum values), corrected © QT interval (QT corrected by Bazett’s formula, along with minimum and maximum values), mean frontal plane QRS electrical axis QT dispersion (d), QTcd, JTd, and JTcd. The presence of pathological Q waves, inverted T waves and ST interval elevation or depression was also recorded. Cycles with artifacts were excluded for the analysis [11]. All methods were carried out in accordance with Good Clinical Practice guidelines and all patients provided written informed consent in accordance with the declaration of Helsinki. Ethical review and approval (ASL1 Avezzano-Sulmona-L’Aquila) were obtained in accordance with local legislation and institutional requirements.

### 2.3. Statistical Analysis

Data were analyzed with STATA/SE software version 16.1 (StataCorp, College Station, TX, USA). The Mann–Whitney U test was used to compare continuous variables while the χ^2^ and Fisher’s exact tests were used as needed for categorical variables. Bivariate correlation (Spearman’s ρ) and binary logistic regression analysis were also performed. All tests were two-tailed and values of *p* < 0.05 were considered statistically significant.

## 3. Results

A total of 123 patients with clinical manifestations, mainly respiratory symptoms/signs, attributable to COVID-19 and laboratory-confirmed SARS-CoV-2 infection and admitted to our COVID-19 unit, after an average of 8 days from symptom onset, were enrolled. As shown in Table 1, the mean age was 73.9 years and 54 (44%) patients were females. Almost 90% of patients displayed at least one CV risk factor and 41 (33%) patients had at least one previous CV event. The patients did not receive any treatment for suspected SARS-CoV-2 infection prior to hospital admission. During the in-hospital stay, the patients were treated with antiplatelet agents or oral anticoagulants as needed, antibiotics other than macrolides and glucocorticoids as indicated by guidelines in effect at the study time. None of the patients were treated with hydroxychloroquine or antivirals known to affect ECG findings during the in-hospital stay. According to the WHO scale for clinical characterization of patients with COVID-19 [12] 57 (46%) patients were in class 4 (not requiring oxygen therapy), 53 (43%) were in class 5 (received oxygen therapy by mask or nasal progs) and 13 (11%) were in class 6 (received oxygen by high flow).

At admission, several ECG abnormalities were recorded, such as ST elevation or depression in 32 (26%) patients, QTc prolongation in 29 (23%) patients, inverted T waves in 28 (23%) patients and pathological Q waves in 19 (15%) patients. Only 10 (8%) had normal ECG findings and all of them recovered from SARS-CoV-2 infection. Of the 123 enrolled patients, 95 (77%) recovered from SARS-CoV-2 infection and were discharged while 28 (23%) died in hospital.

Besides demographic characteristics including older age, laboratory abnormalities, such as higher N/L ratio and clinical features, such as lower SO2 at admission, we observed that several ECG alterations at admission were associated with death (Table 1). When compared to discharged patients, patients who subsequently deceased showed the following abnormalities at the admission ECG: higher HR, QTd, QTcd, longer QTc max, and shorter RR interval, QT interval and QT min. It is important to mention that when focusing on QTc, almost 30% of patients had it prolonged upon admission (≥450 ms in males or ≥460 ms in females) and that no difference was observed in the prevalence of QTc prolongation at baseline in survivors versus deceased patients. Inverted T waves, shown by 23% of patients at admission were more frequently observed in patients that subsequently deceased (46% vs. 16%, *p* < 0.001). Therefore, we performed univariate and multivariate binary logistic regression analysis that is summarized in Table 2.

Given the number of events (death patients N = 28) we selected three variables for multivariate analysis and we opted for unrelated ECG features due to collinearity (e.g., we did not include both HR and RR that are strongly dependent on each other, but only HR). We observed that HR, QTcd and inverted T waves are independently associated with in-hospital death and inverted T waves show the strongest association. This association remained significant even after correcting for the number of CV risk factors at baseline and for the type of CV risk factor at baseline. Based on the existing data [13], we computed a ROC curve attempting to identify a cut-off value of QTcd able to discriminate mortality, but the best performance we could identify was a specificity of 76% with a fairly low sensitivity of 40% for a cut-off value of 39.5 ms.

When correlating demographic, serological features and ECG features at baseline we observed significant associations in the full cohort but not in the subset of deceased patients, most likely due to the low number of subjects (Supplementary Table S1).

As far as discharged patients are concerned, we compared ECG variables recorded at admission and at discharge and observed a reduction in HR (with a consequent increase in the RR interval) and significantly longer QT, QTmax and QTmin (Table 3).

It is interesting to note, that most of the patients showing QTc prolongation, QRS prolongation, or pathological Q waves at admission, still showed these abnormalities at discharge. Conversely, ST interval abnormalities (elevation or depression) and inverted T waves were no longer detectable in half of the patients who showed them at baseline. Supplementary Table S2 shows the comparison of clinical and serological features at admission and at discharge in the 95 survivors.

## 4. Discussion

Cardiac involvement is frequently observed during acute SARS-CoV-2 infection and negatively affects disease prognosis. Our study demonstrated that the majority of patients admitted to the COVID-19 ward with acute SARS-CoV-2 infection displayed at least one ECG abnormality and some of these, such as inverted T waves, were independently associated with in-hospital death regardless of pre-existing CV risk factors.

A broad range of cardiac complications alongside a variety of ECG abnormalities have been described in patients with SARS-CoV-2 infection and linked to different pathogenic mechanisms [4,14,15,16,17,18,19,20,21,22,23,24,25].

Myocardial infarction, arrhythmias, pericarditis and left and/or right ventricular systolic dysfunction may often occur during acute SARS-CoV-2 infection [1]. On ECG assessment, not only abnormalities related to the above clinical pictures but also interval and axis changes, and ST segment and T wave changes are frequently observed [26]. As mentioned, ECG abnormalities during acute SARS-CoV-2 infection may be due to several mechanisms but unfortunately, some aspects of the pathogenic scenario are not yet fully elucidated. Local and systemic inflammation, the so-called “cytokine storm”, direct endothelial or myocardial injury and electrolyte abnormalities are some of the events that may underlie CV manifestations. In order to assess the role of hyperinflammation in developing a cardiac injury, several studies compared patients with COVID-19 developing or not developing cardiac injury and reported that the former display at least one inflammatory marker at a significantly higher level compared to the former [27]. Furthermore, the cytokine storm leads to a disproportionate mobilization of immune cells alongside endothelial dysfunction [28]; there is evidence for cell-mediated cytotoxicity by CD8+ T lymphocytes that migrate into the heart and cause myocardial inflammation [7]. In addition, since SARS-CoV-2 spike protein binds to the membrane-bound form of angiotensin-converting enzyme 2 (ACE2) receptor and ACE2 is expressed in cardiomyocytes, fibroblasts, endothelium, and pericytes, the heart has been proposed as a potential direct target of viral entry [29,30]. The potential cardiotropic effect of SARS-CoV-2 is also supported by the detection of the virus in the interstitial compartment of the myocardium and also in the cardiomyocytes [31,32,33].

Finally, drugs used in earlier phases of the pandemic, such as hydroxychloroquine in combination with azithromycin and some antivirals, had a strong impact on ECG findings, being responsible for the significant prolongation of the QT interval [34]. As evidence of COVID-19 management accrues, international guidelines have been updated and recommend against the above-mentioned compounds due to inefficacy and/or safety issues [35,36]. Therefore, more recent studies allow the evaluation cohorts of hospitalized patients with acute COVID-19 not treated with these compounds, such as our cohort. In this regard, when evaluating QT duration in patients that recovered from SARS-CoV-2 infection we observed a significant increase at discharge compared to admission that, however, was no longer evident when focusing on the QTc. In fact, HR was significantly lower at discharge compared to admission. Importantly, ST interval abnormalities (elevation or depression) and inverted T waves were no longer detectable in half of the patients who showed them at baseline allowing us to speculate an improvement of the subclinical ischemic damage occurring during acute infection. In addition, the significant increase in JTd at discharge may support a persisting direct viral-induced myocardial damage that interferes with conduction despite the acute infection being resolved.

Furthermore, we observed that the presence of inverted T waves, the QTcd and the HR at baseline were independently associated with in-hospital death. The finding on T waves is in line with an American study that evaluated patients with COVID-19 stratified by troponin values [13]. However, in the cohort of Chorin et al., T wave inversions resulted in significantly greater mortality in patients with mild troponin rise while in our cohort the levels of troponin in patients with inverted T waves at baseline were similar to patients that subsequently recovered or deceased.

In addition, Bae et al. demonstrated higher baseline QTcd values in patients who eventually deceased and identified a cut-off value able to discriminate between survival and death with a sensitivity and specificity of over 70% in Korean patients [37]. We also computed a ROC curve for the same purpose but the best performance we could identify was a specificity of 76% with a fairly low sensitivity of 40% for a cut-off value of 39.5 ms.

Several studies reported that patients with pre-existing CV comorbidities, such as hypertension and coronary artery disease that are infected with SARS-CoV-2 have higher mortality compared to those without CV comorbidities [38]. However, other Authors failed to identify this association and this is also the case in our cohort where over 70% of patients had at least one CV risk factor; 33% had at least one previous CV event and the number of deaths was relatively low [13].

Our study displays some limitations including its retrospective nature, the methodological difficulties in measuring/correcting QT intervals, as highlighted in the literature [39] and the lack of assessment of the heart by imaging (e.g., ultrasonography). In addition, our cohort was rather old and age could partially explain why the ECG was abnormal in the vast majority of patients at admission. However, the number of patients deceased during the in-hospital stay was rather low. One unavoidable limitation in the context of an emergency related to an infectious disease is the absence of a pre-hospitalization ECG. In this regard, although it is reasonable to consider abnormalities, such as pathological Q waves as preexisting, it is difficult to distinguish whether the others were from new-onset or COVID-19-related abnormalities.

In conclusion, despite the number of people vaccinated against SARS-CoV-2 increasing, the number of people admitted to hospital due to COVID-19 remains high and the identification of reliable biomarkers to stratify patients at higher risk for severe disease is needed. Our study adds knowledge to this topic by identifying a relationship between baseline ECG abnormalities and COVID-19 outcomes, such as in-hospital death. These findings may be of particular relevance in clinical settings with limited access to advanced techniques, such as CMR and could help improve the outcomes of patients with cardiac involvement related to SARS-CoV-2 infection. We should discuss the results and how they can be interpreted from the perspective of previous studies and the working hypotheses. The findings and their implications should be discussed in the broadest context possible. Future research directions may also be highlighted.

## Figures and Tables

**Table 1:** Baseline characteristics of the entire patient cohort (N = 123) and comparison of baseline characteristics according to the outcome (N = 95 discharged vs. N = 28 deceased).

	All		Discharged		Deceased		*p* Value
	**N**	**%**	**N**	**%**	**N**	**%**	
Total number of patients	123	100	95	77	28	23	na
Female gender	54	44	38	40	16	57	0.108
Fever	23	19	15	16	8	28	0.13
CV risk factors							0.07
*0*	36	29	23	24	13	46	
*1–2*	73	60	60	63	13	46	
*≥3*	14	11	12	13	2	8	
Previous CV event	41	33	30	31	11	39	0.45
Normal ECG	10	8	10	100	0	0	na
QTc prolongation *	29	23	19	20	10	36	0.08
QRS prolongation ^†^	16	13	11	12	5	18	0.38
Pathological Q wave	19	15	14	15	5	18	0.69
ST elevation or depression	32	26	21	22	11	39	0.07
Inverted T wave	28	23	15	16	13	46	<0.001
	**Median**	**range**	**Median**	**range**	**Median**	**range**	
Non-CV comorbidities	1	0–6	1	0–6	1	0–4	0.31
	**Mean**	**SD**	**Mean**	**SD**	**Mean**	**SD**	
Symptoms to admission (days)	8.4	10	7.8	7.1	9.7	17.3	0.05
Age	73.9	15.5	70.4	15.4	85.9	8.4	<0.001
BMI	27.3	4.0	27.8	3.9	25.4	3.8	0.005
N/L ratio	7.7	7.7	5.8	4.8	12.8	11.1	<0.001
ESR (mm/1 h)	59.6	27.0	57.3	24.6	71.2	35.9	0.28
CRP (mg/L)	17.1	57.2	18.2	64.0	13.4	19.9	0.015
Ferritin	529.2	547.8	478.1	441.6	748.5	853.5	0.506
Procalcitonin	4.0	30.6	4.1	33.9	3.3	5.1	<0.001
Troponin	130.3	349.2	86.8	304.2	274.1	446.6	<0.001
SO2 in AA (%)	93.9	4.2	94.9	2.4	90.8	6.4	0.006
PaO2/FiO2	268.5	92.4	279.4	93.0	242.3	88.1	0.159
HR (b/minute)	81.9	21.1	76.7	14.6	100.4	28.8	<0.001
RR interval (ms)	755.4	195.9	807.6	165.2	580.1	191,9	<0.001
QRS (ms)	107.32	84.1	109.7	94.7	99.1	23.2	0.51
QT (ms)	383.07	47.4	387.4	36.7	368.2	72.3	0.04
QTmax (ms)	394.9	49.9	397.2	39.2	387.4	76.9	0.348
QTmin (ms)	369.9	49.2	377.3	40.6	344.2	66.1	0.003
QTc (ms)	435.6	46.5	432.1	36.9	447.8	70.1	0.980
QTc max (ms)	450.5	51.1	442.9	39.5	476.56	74.3	0.04
QTc min (ms)	421.2	46.4	420.7	41.3	422.8	61.8	0.98
QTd (ms)	26.4	30.7	21.7	27.3	42.4	36.7	0.007
QTcd	30.7	35.1	23.9	29.3	53.8	42.9	<0.001
JTd	284.18	46.9	288.5	36.2	269.9	71.1	0.108
JTcd	334.0	55.8	329.1	48.4	350.2	74.2	0.122

CV, cardiovascular; N/L, neutrophil-to-lymphocyte; ESR, erythrocyte sedimentation rate; CRP, C reactive protein; SO2, oxygen saturation; PAO2/FiO2, pressure of arterial oxygen to fractional inspired oxygen concentration; HR, heart rate; b, beats; c, corrected; d, dispersion; min, minumum; max, maximum; na, not applicable * ≥450 ms in male or QTc interval ≥460 ms in female ^†^ >120 ms.

**Table 2:** Binary logistic regression analysis shows the association between ECG parameters at baseline and death. Due to the number of events (death N = 28), three unrelated variables were included in the multivariate analysis.

	Univariate			Multivariate		
	OR	95% CI	*p* Value	OR	95% CI	*p* Value
HR (b/minute)	1.07	1.03–1.10	<0.001	1.09	1.04–1.14	<0.001
RR interval (ms)	0.99	0.98–0.99	<0.001	-	-	-
QRS (ms)	0.99	0.98–1.01	0.64	-	-	-
QT (ms)	0.99	0.98–1.01	0.08	-	-	-
QTmax (ms)	0.99	0.99–1.01	0.39	-	-	-
QTmin (ms)	0.98	0.97–0.99	0.006	-	-	-
QTc (ms)	1.01	0.99–1.02	0.14	-	-	-
QTc prolongation	2.35	0.91–6.07	0.08	-	-	-
QTc max	1.01	1.004–1.02	0.006	-	-	-
QTc min	1.01	0.99–1.01	0.846	-	-	-
QTd (ms)	1.02	1.01–1.04	0.005	-	-	-
QTcd	1.02	1.01–1.04	<0.001	1.03	1.01–1.05	0.003
JTd	0.99	0.98–1.00	0.08	-	-	-
JTcd	1.00	0.99–1.01	0.10	-	-	-
Pathological Q wave	1.29	0.41–4.00	0.66	-	-	-
ST elevation or depression	2.43	0.95–6.17	0.06	-	-	-
Inverted T wave	2.67	1.57–4.57	<0.001	3.29	1.54–7.05	0.002

HR, heart rate; b, beats; c, corrected; d, dispersion; min, minumum; max, maximum; OR, odds ratio; CI, confidence interval.

**Table 3:** Comparison of electrocardiographic findings ad admission and at discharge in the 95 patients that recovered from SARS-CoV-2 infection.

	Admission	Discharge	*p* Value
HR (b/minute)	76.7 ± 14.6	68.9 ± 13.6	<0.001
RR interval (ms)	807.6 ± 165.2	922.8 ± 350.6	<0.001
QRS (ms)	109.7 ± 94.7	115.5 ±107.4	0.68
QT (ms)	387.4 ± 36.7	413.2 ± 49.6	<0.001
QTmax (ms)	397.2 ± 39.2	422.5 ± 51.1	0.001
QTmin (ms)	377.3 ± 40.6	401.8 ±52.3	0.002
QTc (ms)	432.1 ± 36.9	433.5 ± 39.2	0.50
QTc max	442.9 ± 39.5	443.3 ± 40.9	0.28
QTc min	420.7 ± 41.3	421.1 ± 37.8	0.56
QTd (ms)	21.7 ± 27.3	20.3 ± 28.8	0.46
QTcd	23.9 ± 29.3	22.2 ±47.5	0.40
JTd	288.5 ± 36.2	310.4 ± 47.2	0.001
JTcd	329.1 ± 48.4	335.7 ± 44.8	0.95
QTc prolongation *	19 (20)	16 (17)	0.574
QRS prolongation ^†^	11 (12)	8 (8)	0.47
Pathological Q wave	14 (15)	12 (13)	0.673
ST elevation or depression	21 (22)	11 (12)	0.05
Inverted T wave	15 (16)	8 (8)	0.12

Values are shown as mean ± standard deviation or number of patients (%) and *p* values are calculated with the Wilcoxon matched pairs test or the Chi-square test, respectively. * ≥450 ms in male or QTc interval ≥460 ms in female; ^†^ >120 ms; HR, heart rate; b, beats; c, corrected; d, dispersion; min, minumum; max, maximum.

## Data Availability

All relevant data are included in the main manuscript and supplementary material.

## Supplementary Materials

The following supporting information can be downloaded at: https://www.mdpi.com/article/10.3390/jcm11175248/s1, Table S1: Linear bivariate correlation between demographic/serological features and electrocardiographic findings at baseline; Table S2: Comparison of clinical and serological features at admission and at discharge in the 95 patients that recovered from SARS-CoV-2 infection.
