# Supplementary material for: Electrocardiographic Abnormalities in Hospitalized Patients with COVID-19 and the Associations with Clinical Outcome
Source: J Clin Med. 2022 Sep 5;11(17):5248. doi: 10.3390/jcm11175248 (PMC9457256; doi:10.3390/jcm11175248)
Supplement: Supplementary file 1 [file jcm-11-05248-s001.zip › jcm-1835975-supplementary.pdf]

Supplementary Materials

Supplementary Table S1. Linear bivariate correlation between demographic/serological features and electrocardiographic findings at baseline.

	All Patients									
	Age		CRP		Ferritin		Procalcitonin		BNP	
	CC	*p* Value	CC	*p* Value	CC	*p* Value	CC	*p* Value	CC	*p* Value
HR										
RR	-0.39	<0.0001	-0.21	0.034	-0.33	0.29	-0.47	<0.0001	-0.29	0.005
PR	-	ns	-	ns	-	ns	-	ns	-	ns
QRS	-	ns	-	ns	-	ns	-	ns	-	ns
QT	-	ns	-	ns	-	ns	-	ns	-	ns
QTc	0.27	0.004	0.28	0.003	-	ns	0.37	<0.0001	0.22	0.032
QT max	-	ns	0.28	0.004	-	ns	0.43	<0.0001	-	ns
QT min	-	ns	0.24	0.013	-	ns	0.24	0.019	-	ns
QTD	-	ns	-	ns	-0.22	0.048	-	ns	-	ns
QTc max	0.246	0.009	-	ns	-	ns	-	ns	0.254	0.013
QTc min	0.297	0.002	-	ns	-	ns	-	ns	-	ns
QTcD	-	ns	-	ns	-	ns	0.254	0.014	-	ns
JTD	-	ns	-	ns	-	ns	-	ns	-	ns
JTcD	0.315	0.001	0.240	0.014	-	ns	0.312	0.002	-	ns

CC, Spearman's rho correlation coefficient; HR, heart rate; CRP, C reactive protein; BNP, brain natriuretic peptide

Supplementary Table S2. Comparison of clinical and serological features at admission and at discharge in the 95 patients that recovered from SARS-CoV-2 infection.

	Admission	Discharge	*p* Value
N/L ratio	5.7±4.8	4.4±6.5	<0.001
ESR (mm/1h)	57.4±24.5	62.7±34.5	0.180
CRP (mg/L)	18.2±64	1.55±2.5	<0.001
Ferritin (ng/mL)	478.2±441	356.4±269	<0.001
Procalcitonin	4.1±33.8	0.72±3.2	<0.001
SO2 in AA (%)	94.9±2.4	95.7±2.1	0.043
PaO2/FiO2	279±92.9	337.5±85.9	0.214

Values are shown as mean ± standard deviation. HR, heart rate.
